# Supplementary material for: Identification of photoperiod-induced specific miRNAs in the adrenal glands of Sunite sheep (Ovis aries)
Source: Front Vet Sci. 2022 Jul 22;9:888207. doi: 10.3389/fvets.2022.888207 (PMC9354845; doi:10.3389/fvets.2022.888207)
Supplement: Supplementary file 1 [file Data_Sheet_1.ZIP › Supplementary file/miRDeep2 file.zip!/miRDeep2 file/Know_Structure/SP42A3/oar-mir-411b.pdf]

miRBase precursor : oar-mir-411b
Total read count : 1341
oar-miR-411b-5p read count : 1320
oar-miR-411b-3p read count : 21
remaining reads : 0

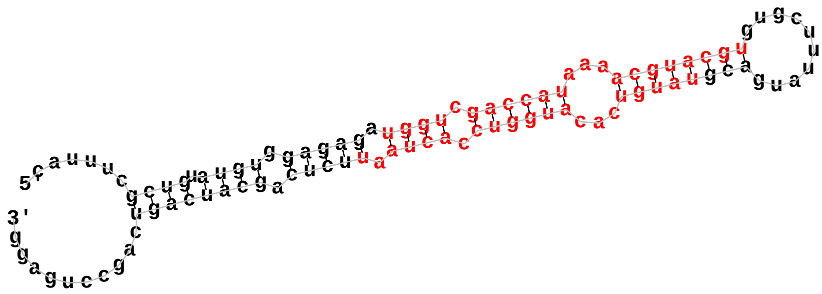

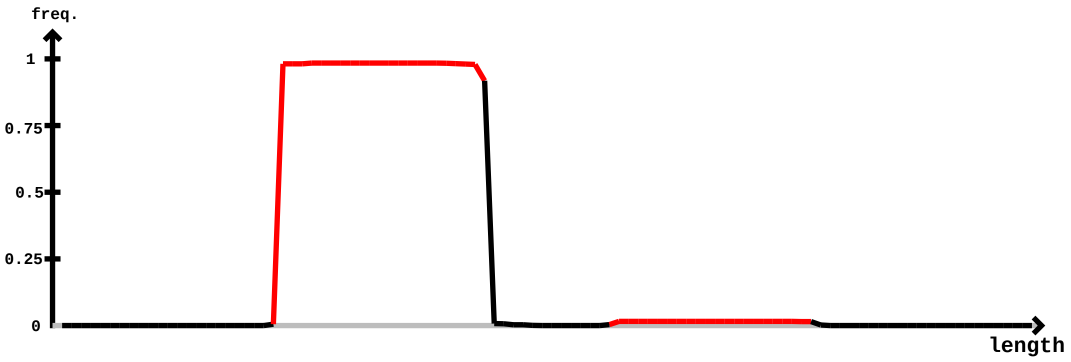


```
                                                         oar-miR-411b-3p
                     oar-miR-411b-5p
5'- cauuucgcuguauguggagagauggucgaccauaaaacguacgugugcuuuaugacguaugucacaugguccacuaauucucagcaucagucagccugagg -3'  exp
    ......((((.((((.(((((.((((.((((((...((((((((..........))))))))...)))))).)))).))))).))))))))..........   reads   mm   sample
    .....................auggucgaccauaaaacguacg..........................................................   4       0    seq
    .....................auggucgaccauaaaacguacgu.........................................................   3       0    seq
    ......................uggucgaccauaaaacg..............................................................   1       0    seq
    ......................uggucgaccauaaaacgu.............................................................   2       0    seq
    ......................uggucgaccauaaaacgua............................................................   2       0    seq
    ......................uggucgaccauaaaacguac...........................................................   2       0    seq
    ......................uggucgaccauaaaacguacg..........................................................   78      0    seq
    ......................uggucgaccauaaaacguacgu.........................................................   1218    0    seq
    ......................uggucgaccauaaaacguacgug........................................................   1       0    seq
    ......................uggucgaccauaaaacguacgugu.......................................................   5       0    seq
    .........................ucgaccauaaaacguacgugugc.....................................................   3       0    seq
    .........................ucgaccauaaaacguacgugugcu....................................................   1       0    seq
    ........................................................guaugucacaugguccacua.........................   1       0    seq
    ........................................................guaugucacaugguccacuaau.......................   4       0    seq
    .........................................................uaugucacaugguccacuaau.......................   13      0    seq
    .........................................................uaugucacaugguccacuaauu......................   3       0    seq
```
